# Precipitation, EMS use, and time to presentation of Acute Ischemic stroke in the get with the guidelines-stroke registry

**DOI:** 10.1016/j.jstrokecerebrovasdis.2025.108460

**Published:** 2025-09-27

**Authors:** Marisa Berner, Mathew Reeves, George S. Usmanov, Kevin N. Sheth, Amar Dhand

**Affiliations:** a Harvard Medical School, 25 Shattuck Street, Boston, MA 02115, USA; b Department of Neurology, Brigham & Women’s Hospital, 65 Landsdowne Street, Cambridge, MA 02139, USA; c Department of Epidemiology and Biostatistics, Michigan State University, 909 Wilson Road Room B601, East Lansing, MI 48824, USA; d Department of Neurology & Neurosurgery, Yale School of Medicine, 800 Howard Avenue, New Haven, CT 06519, USA; e Yale Center for Brain & Mind Health, 333 Cedar Street, New Haven, CT 06510, USA; f Network Science Institute, Northeastern University, 177 Huntington Avenue, #1010, Boston, MA 02115, USA

**Keywords:** Ischemic stroke, Time-to-Treatment, Registries, Weather, Emergency medical services

## Abstract

**Background:**

Delayed presentation to the hospital is a barrier for delivering acute stroke treatments. The effects of weather on delay are unstudied. We examined the relationship between precipitation and time to arrival nationwide.

**Methods:**

We studied patients with acute ischemic stroke who presented at a Get With the Guidelines^®^-Stroke hospital from 2010–2019. The exposure variable was total daily precipitation on the day of presentation, obtained from the National Oceanic and Atmospheric Administration, and categorized as none (0 inches/day), mild (>0–1 inch/day), and major (>1 inch/day). The primary outcome was time to hospital arrival after stroke (in minutes). We conducted multivariate regression analysis, including analysis of interaction between precipitation level and EMS usage.

**Results:**

In ~2.7 million patients, 50.7% were female and 38.6% were ≥75 years old. Median time to hospital arrival was 212 minutes in no precipitation, 219 minutes in mild precipitation, and 223 minutes in major precipitation. In adjusted analyses, compared to no precipitation, mild precipitation was associated with 4.63 minutes of delay [95% CI: (2.77, 6.49)]. Major precipitation was associated with 7.69 minutes of delay [95% CI: (2.86, 12.52)]. EMS usage improved arrival time overall, and there was an interaction with mild precipitation (−6.09 minutes [95% CI: (−9.79 to −2.39)]). However, there was no interaction with major precipitation (−2.72 minutes [95% CI: (−12.38 to 6.93)]).

**Conclusion:**

Precipitation was associated with delayed presentation to the hospital in acute stroke. Increased frequency of extreme weather calls for developing EMS strategies and infrastructure to support climate-ready stroke systems of care.

## Introduction

Reducing prehospital delay is one of the greatest outstanding needs in acute stroke treatment.^1,2,3^ Although rapid arrival is critical for patients who receive reperfusion therapies, all patients benefit from early assessment and treatment.^4,5,6^ Consequently, the prehospital time window is arguably the most critical time window left to optimize in stroke systems of care.

There are a multitude of factors that affect prehospital arrival time including age, gender, stroke severity, co-morbidities, and EMS.^7^ While a significant amount of attention has been appropriately dedicated to individual risk factors, socio-ecological factors also require consideration to effectively minimize levels of risk and barriers to treatment.^8,9^ Such factors include time of day during the stroke event and method of transportation. Persons who experience symptoms during the day, for example, are significantly more likely to arrive to the hospital in under four hours^10^. Similarly, helicopter transportation has been found to significantly decrease the delays for stroke patients, particularly in more rural areas.^11^ With ground transportation, significant barriers include traffic patterns, particularly in relation to rush hours and time of day.^12^ Other socio-ecological factors that have gained increasing recognition social vulnerability of a neighborhood, transportation infrastructure, and ambulance coverage – all of which reveal systemic weaknesses.^13,14^

One factor in prehospital arrival times that has received less attention is weather conditions. Extreme weather may increase the number of emergency department presentations and pose challenges to getting to the hospital.^15^ Adverse weather events and air quality have been shown to be a significant risk factor for stroke risk within a community.^1,16,17^ Moreover, older and frail patients, who are most vulnerable to stroke, may be more susceptible to delayed arrival due to weather events.^18^ Additionally, efforts to accelerate EMS response have been implemented with the consideration that weather patterns are in line with historical trends – an assumption that, due to climate change, is becoming increasingly fallible.^19^ Projections of climate change have further predicted that stroke incidence will increase in line with extreme weather and temperature events, with the ecological changes making it progressively more difficult for first responders to respond quickly.^20,21^ Therefore, there is a gap and a need in understanding climate change effects on stroke systems of care.

We examined the association of daily total precipitation with time to presentation for acute stroke in a nationwide study over 9 years. Our hypotheses were that 1) the presence of precipitation leads to a delay in time to hospital arrival in acute ischemic stroke, and 2) the use of EMS mitigates this delay.

## Methods

### Data source

We used data from the American Heart Association’s Get With The Guidelines (GWTG) Stroke Registry, a prospective, hospital-based voluntary national registry database. Over 9,000,000 patients across approximately 2,800 hospitals have been registered since 2003. We used data from 2010–2019. We studied variables including participant’s onset to time to hospital arrival after stroke, date of stroke, location of stroke event, NIH stroke score (NIHSS), age, race, ethnic identity, gender, and zip code.

We used data on daily precipitation from an online repository of historical precipitation data sourced from the National Oceanic and Atmospheric Administration.^22^ We used ten years of daily precipitation data for US zip codes. This included 3,650 data points per zip code for ~40,000 US zip codes.

### Standard protocol approvals, registrations, and patient consents

IQVIA (Parsippany, New Jersey) serves as the data collection and coordination center for the GWTG Stroke Registry. Each participating hospital received either human research approval to enroll cases without individual patient consent under the common rule, or a waiver of authorization and exemption from subsequent review by their institutional review board (IRB). Advarra, the IRB for the American Heart Association, determined that this study is exempt from IRB oversight.

### Data availability statement

Due to the sensitive nature of data collected for this study, access to the Get With The Guidelines (GWTG) Stroke Registry dataset is available upon request from the American Heart Association Quality Programs Research Publications at qualityresearch@heart.org.

### Study population

Between 2010 and 2019, we identified 2,663,493 patients with an acute ischemic stroke who came to a GWTG hospital within 48 hours of onset. We excluded patients who arrived after 48 hours because they are unlikely to receive reperfusion therapy. Figure S1 illustrates the flow chart of patients included in our analysis, and how we handled missing data. We excluded patients who were missing home zip codes, were in a hospital at the time of stroke onset, or did not have complete demographic data.

### Exposure variables and study outcomes

To combine the two datasets and link precipitation information to the day of the stroke for each participant, we organized precipitation data into three columns: date, zip code, and precipitation occurring that day. Then, we matched to the GWTG dataset based on participants’ date of admission and zip code in both datasets. The primary exposure variables were the total daily precipitation (recorded in inches/day) and the use of EMS. We categorized precipitation from the National Oceanic and Atmospheric Administration and divided into three categories: No precipitation (0 inch/day), mild precipitation (>0–1 inch/day), and major precipitation (>1 inch/day). We chose cut points to reflect meaningful contrasts between no precipitation, light rainfall unlikely to affect mobility, and heavy precipitation events that could plausibly alter health behaviors and access to care. EMS was categorized as either using EMS or not using EMS. We included mobile stroke units in the using EMS category. The covariates, based on prior literature, were participants’ age, race, ethnicity, gender, NIHSS, rural/urban setting (according to USDA Economic Research Service), and region of the United States.

The primary outcome of interest was time to hospital arrival after stroke (in minutes). The Duke Clinical Research Institute calculated the variable, onset to arrival time (OTA_time), from the GWTG-Stroke database. In 80% of cases, this is defined as last known well to arrival to the hospital. In 20% of cases where last known well time was missing, less than zero, or greater than 7 days, this variable was calculated as time from symptom onset to arrival to the hospital.

### Statistical analysis

We used the American Heart Association Precision Medicine Platform (https://precision.heart.org) for data analysis. Following descriptive analyses of all key variables we completed a multivariable linear regression for the primary outcome of onset to arrival time in minutes. This regression included the exposure variables (precipitation as 0 inch/day, >0–1 inch/day, or ≥1 inch/day and EMS use) and covariates (age, NIHSS score, sex, region of the United States, rural/urban setting, race, and ethnicity). After generating the main effects model we tested an interaction term between the 2 primary exposure variables of interest (precipitation and EMS use). Finally, we completed a sensitivity analysis with fixed effects of State in place of Region of Country. We followed the STROBE reporting guideline (Table S1). All analyses were conducted using RStudio.

## Results

Of the 2,663,493 persons with acute ischemic stroke, 50.7% were female and the median age was 71 years (Interquartile range, IQR = 21). Further descriptives are provided in Table 1. Median level of precipitation was 0.0 inches (IQR = 0.04). Median time to hospital arrival after stroke was 226 minutes (IQR = 517). Encounters with mobile stroke units (N=2004) were not prevalent to be considered as a separate subgroup from traditional EMS use (N=1,230,105). Therefore, they were included within the Used EMS category.

In terms of the relationship between precipitation and time to hospital arrival, on days with no precipitation, the median arrival time was 212 minutes (IQR = 496). On days with mild precipitation (0–1 inch), the median arrival time was 219 minutes (IQR = 504). On days with major precipitation (>1 inch), the median arrival time was 223 minutes (IQR = 511). In Table 2, these times are stratified by EMS usage. It shows markedly faster median arrival times for those who used EMS (126 minutes versus 313 minutes on days with no precipitation). There was also a *monotonic* increase of 3 minutes between no precipitation to mild, and 3 minutes between mild to major. In contrast, those who did not use EMS showed a *non-monotonic pattern* with an increase of 12 minutes from no precipitation to mild, and 5 minutes between mild to major.

In the multivariable linear regression (Table 3), mild precipitation was independently associated with 4.63 minutes [95% CI: (2.77, 6.49)] of delay. Major precipitation was independently associated with 7.69 minutes [95% CI: (2.86, 12.52)] of delay. The use of EMS was associated with a reduction in delay of 190.89 minutes [95% CI (−192.67, −189.12)].

**Fig. 1.**
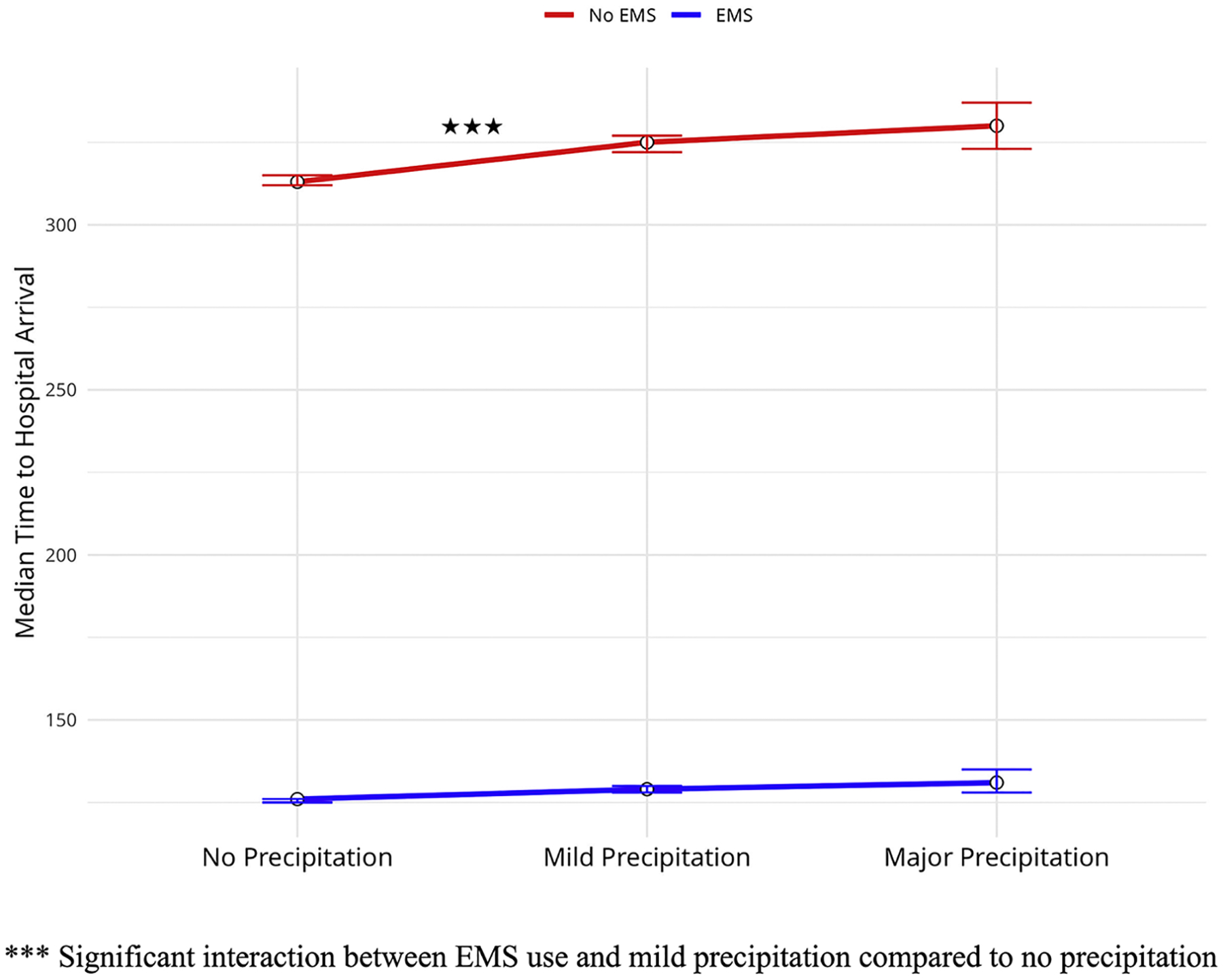
Arrival Time to Hospital Across Varying Precipitation in Persons Who Do Not Use EMS versus Persons Who Use EMS.

In the multivariable linear regression with interactions (Table 4), we examined the relationship of precipitation to arrival time as modified by use of EMS. The use of EMS in conditions of mild precipitation was associated with a significant decrease in arrival time to the hospital by an average of −6.09 minutes [95% CI: (−9.79 to −2.39)], compared to conditions with no precipitation. However, the association between EMS use and arrival time during major precipitation events was not statistically significant, with a beta of −2.72 minutes [95% CI: (−12.38 to 6.93)], indicating no interaction between EMS use and major precipitation. Fig. 1 summarizes this pattern.

As a sensitivity analysis, we substituted US State instead of the 4 Regions of the US as a fixed effect in a multivariable linear regression (Table S2). The analysis included 46 individual US States. States absent in the dataset included Connecticut, Maine, New Hampshire, and Vermont. We completed this analysis to achieve greater control for the variation in precipitation by location. We found similar results to our main models. Mild precipitation was independently associated with 3.30 minutes [95% CI: (1.42, 5.19)] of delay. Major precipitation was independently associated with 8.03 minutes [95% CI: (3.20, 12.88)] of delay. The use of EMS was associated with a reduction of delay by 190.65 minutes [95% CI: (−192.42, −188.87)]. Finally, we completed a log transformation of the time to arrival variable and found similar results (Table S3).

## Discussion

In this nationwide cohort study, we examined the association between daily total precipitation and the time to hospital arrival after acute ischemic stroke. We found that the level of precipitation on the day of presentation had a significant association with arrival time. Mild precipitation was associated with 4.63 minutes of delay, and major precipitation was associated with 7.69 minutes of delay. As expected, EMS use was associated with a decrease in delay; patients that used EMS arrived on average 190.89 minutes sooner than those who arrived by private transport. The effect of precipitation depended on EMS use, with those who used EMS gaining 6.09 minutes during days with mild precipitation, while the effect of major precipitation was not modified by EMS use. Our result supported the hypothesis that precipitation was related to prehospital delay, and partially supported the hypothesis of EMS mitigating this effect.

These findings contribute to the growing literature on the ways in which weather may impact access to care during health emergencies, specifically the role of weather severity on stroke systems of care.^23–25^ In the stroke literature, researchers using GWTG-stroke have shown a relationship between temperature and precipitation on in-hospital mortality, discharge home, and independent ambulation at discharge after stroke. Specifically, warmer and wetter weather conditions were independently associated with mortality and discharge disposition, although time to arrival was not studied.^26^ Researchers also found that weather patterns may lengthen the length of time it takes for a patient to receive medical care outside of stroke events^27^ Other researchers have found that temperatures relate to ambulance dispatches for stroke, which in turn, affect arrival time.^28^

The mechanisms driving these findings of weather-related delays deserve further study. Potential mechanisms include psychological hesitation to seek care during adverse weather, traffic congestion, accidents, and the overextension of emergency medical services.^29–31^ These factors could contribute to delays in hospital arrival and worsened stroke outcomes during severe weather.^32,33^ For example, heavy rainfall or snow may cause individuals to delay leaving their homes, either due to fear of hazardous travel conditions or uncertainty about the severity of their symptoms. At the system level, the EMS-moderation effect appears to disappear during major precipitation (>1 inch). This is when traffic slowdowns and capacity strain within EMS systems blunt the usual benefits of timely emergency response, leading to prolonged time to treatment. Understanding how both individual behaviors, such as protective measures and delays in help-seeking, and systemic issues, like traffic and emergency response capacity, impact care delivery is crucial. Additionally, applying a climate-focused lens to stroke systems of care is necessary to build resilience, improve EMS capacity during weather disruptions, and ensure timely access to care.^34,35^ Future efforts should prioritize preparing healthcare infrastructure and emergency services to handle the growing frequency of extreme weather events.^36,37^

The findings from our study have several specific implications for climate-resilient health system planning. First, protocols for EMS systems during hazardous weather should be optimized to minimize prehospital delays. This may require precision weather forecasting; some industries are already using drones and other emerging technologies to capture real-time weather information and plan responses accordingly. Second, adaptive EMS staffing adjusted for weather may be crucial to maintaining quality care. Finally, our study highlights the need for future research on the relationship between weather and stroke care, including systematic analyses of temperature, wind, snow, ice, humidity, visibility, and extreme weather events. Such work should also evaluate the impact of weather-related delays on key clinical outcomes, including eligibility for thrombolytic therapy and 3-month functional outcomes, to ensure that adaptation strategies ultimately translate into better patient care.

There are limitations to this study. A zip code-based daily weather analysis may include weather patterns that do not affect the patient’s specific location or exact time of the acute stroke. We also did not account for additional weather concerns such as wind speed, road conditions, buffer zones, major rivers, geographic variation, and the presence of ice, snow, thunderstorms, or hail. These are important factors to study in future research. Residual measured and unmeasured confounding may additionally have affected the findings of the study. Low statistical precision may have affected the moderation analysis. Finally, analyses conducted do not provide insights into the long-term wellbeing of stroke survivors after their event. However, studies have shown strong correlations between arrival time and functional outcomes at 3 months.^38^

In conclusion, our results indicate that precipitation was associated with prehospital delay for patients experiencing acute stroke. The use of EMS partially mitigated this relationship. Further research is needed in understanding the effect of broader weather conditions and their mechanisms to create better strategies to improve access to care. As climate change and extreme weather events become more prevalent, such research is needed to create climate-ready stroke systems of care.

## Supplementary Material

1

Supplementary material associated with this article can be found, in the online version, at doi:10.1016/j.jstrokecerebrovasdis.2025.108460.

## Figures and Tables

**Table 1:** Participant Demographics.

Demographic Category	Number	Percentage
Sex		
Female	1,351,421	50.7
Male	1,312,072	49.2
Race		
White	1,944,984	73.0
Black	465,679	17.5
Asian	81,270	3.1
Other	171,560	6.4
Ethnicity		
Hispanic	205,468	7.7
Not Hispanic	2,457,067	92.2
Region of the United States^†^		
South	841,098	31.6
Northeast	331,001	12.4
Midwest	434,403	16.3
West	375,230	14.1
Missing	681,761	25.6
Rural-urban classification		
Urban	2,073,002	77.8
Rural	382,102	14.3
Missing	208,389	7.8
Stroke Severity (NIHSS)		
Mild (> 4)	1,333,606	50.1
Moderate (5–15)	615,200	23.1
Severe (16+)_	307,954	11.6
Missing	406,833	15.2
Used EMS	1,232,364	46.2
Did Not Use EMS	1,410,555	53.0

† 46 individual states including Alaska and Hawaii were included and organized into 4 regions. Alaska and Hawaii were categorized as “West”.

**Table 2:** Arrival Time to Hospital Divided by Precipitation and EMS.

	Count	Median (Minutes)	Interquartile Range
No EMS			
No Precipitation	383,411	313	588
Mild Precipitation	163,397	325	589
Major Precipitation	17,513	330	599
EMS			
No Precipitation	423,762	126	352
Mild Precipitation	182,765	129	358
Major Precipitation	19,731	131	369

**Table 3:** Linear Regression Model of Daily Precipitation, EMS Use, and Time to Hospital Arrival After Stroke.

Variables (N=1,190,698)	Estimate	Confidence Interval (95%)
Constant	475.00***	(469.90, 480.11)
Southern Region (ref)	–	–
Northeastern Region	−7.25***	(−9.69, −4.81)
Midwestern Region	−1.67	(−3.88, 0.53)
Western Region	−18.13***	(−20.53, −15.73)
Rural Setting (ref)	–	–
Urban Setting	−38.75***	(−41.11, −36.38)
Age (In Decades)	3.20***	(2.60, 3.79)
NIHSS (Severe) (ref)	–	–
NIHSS (Moderate)	36.99***	(34.36, 39.62)
NIHSS (Mild)	43.74***	(41.27, 46.22)
Male (ref)	–	–
Female	−1.73***	(−3.42, −0.04)
White or Caucasian (ref)	–	–
Black or African American	51.39***	(49.07, 53.71)
Asian or Asian American	46.81***	(41.84, 51.78)
Not Hispanic (ref)	–	–
Hispanic	24.96***	(21.30, 28.61)
EMS Not Used (ref)	–	–
EMS Used	−190.89***	(−192.67, −189.12)
No Precipitation (ref)	–	–
Mild Precipitation	4.63***	(2.77, 6.49)
Major Precipitation	7.69***	(2.86, 12.52)

Note:

** indicates significance at p < .05 and

*** indicates significance at p < .001.

**Table 4:** Linear Regression Model of Precipitation and Time to Hospital Arrival After Stroke with Interaction with EMS Usage.

Variables (N = 1,190,698)	Estimate	Confidence Interval (95%)
Constant	473.97***	(468.82, 479.11)
Southern Region (ref)	–	–
Northeastern Region	−7.24***	(−9.68, −4.80)
Midwestern Region	−1.67	(−3.88, 0.53)
Western Region	−18.15***	(−20.56, −15.75)
Rural Setting (ref)	–	–
Urban Setting	−38.73	(−41.10, −36.36)
Age (In Decades)	3.20***	(2.60, 3.80)
NIHSS (Severe) (ref)	–	–
NIHSS (Moderate)	37.01***	(34.38, 39.64)
NIHSS (Mild)	43.77***	(41.30, 46.25)
Male (ref)	–	–
Female	−1.73**	(−3.42, −0.04)
White or Caucasian (ref)	–	–
Black or African American	51.39***	(49.07, 53.71)
Asian or Asian American	46.79***	(41.82, 51.76)
Not Hispanic (ref)	–	–
Hispanic	25.00***	(21.35, 28.66)
EMS Not Used (ref)	–	–
EMS Used	−189.04***	(−191.15, −186.93)
No Precipitation (ref)	–	–
Mild Precipitation	7.84***	(5.14, 10.53)
Major Precipitation	9.12**	(2.09, 16.15)
EMS Used * No Precipitation (ref)	–	–
EMS Used * Mild Precipitation	−6.09***	(−9.79, −2.39)
EMS Used * Major Precipitation	−2.72	(−12.38, 6.93)

Note:

** indicates significance at p < .05 and

*** indicates significance at p < .001.
